# Editorial

**Published:** 1900-08-15

**Authors:** 


### EDITORIAL.

THE annual meeting of the National Associations were held at Old Point Comfort, Virginia, July 10th to 16th. Both meetings were well attended. For some unexplained reason the papers contributed were not up to the grade of the previous year, neither were so many offered Possibly the Paris meeting had something to do with the lack in this respect. Steps have been taken to insure a more complete report by the sections for the future. Each Section has appointed a commission to make original researches, or at least compile the activities of the year into a well-digested report to be read at the annual meeting.

Dr. G. V. Black of Chicago, was elected President of the National Dental Association, and the next meeting will be held at Milwaukee, the first Tuesday in August, 1901.

THE meeting of the National Association of Dental Faculties was harmonious and uneventful. By a great effort the entrance requirements for students entering dental schools two years hence will be increased to those of the second year in a high school We ought to be duly grateful for small favors and we should be hopeful, were we not aware of the fact that this resolution may be reconsidered and revoked before it can be put into practice. There is no adequate reason why this powerful organization can not for once and all time make high school graduation the requirement for admission to its schools. No one questions the desirability of ultimately reaching this standard If all schools are compelled to adopt it at the same time, where would be the harm?

Dr. B. Holly Smith was elected President, and the next meeting will be held at Milwaukee in connection with the National Dental Association.

---

Dr. Alphonse Irwin, in reporting the proceedings of the Section on Stomatology of the American Medical Association, for the Southern Dental Society of New Jersey, in speaking of the discussion on dental education, complains that "gifts of mechanical genius or scientific acuteness of intellect" in the student were not considered of sufficient importance to be even mentioned. He believes that "successful training is subjective as well as objective." And he wants to know how a Bonwill or Webb would be educated by the stomatologists. Subjective training or the training of the individual is ideal, but alas! impracticable under existing conditions. Hand-picked fruit may be choice, but it is also expensive. Besides, geniuses are not the result of a system of pedagogics, and can never be taken into account in educational plans for the masses. They may not be advanced as rapidly when compelled to develop or train with the average students, but they are seldom injured by this association. The genius is a rare person, and he is so scarce that no college has as yet been organized to take charge of his training. The school organized for this purpose will need a large endowment, as a large faculty of self-sacrificing teaches will be necessary, to say nothing of the material equipment. The dental educational system must be planned for the average man, and private individuals with benevolent dispositions must look after the specialists.

---

The *Items of Interest* of this month contains a good sketch of the National meetings and some very creditable

"snap shot" pictures of some of the prominent members in attendance. Those of Drs. Taft and Black are particularly good. Dr. Ottolengui has, by his excellent report, again demonstrated his journalistic capacity.

